# Summer, sun and sepsis—The influence of outside temperature on nosocomial bloodstream infections: A cohort study and review of the literature

**DOI:** 10.1371/journal.pone.0234656

**Published:** 2020-06-19

**Authors:** Frank Schwab, Petra Gastmeier, Peter Hoffmann, Elisabeth Meyer

**Affiliations:** 1 Charité–Universitätsmedizin Berlin Corporate Member of Freie Universität Berlin, Humboldt-Universität zu Berlin, and Berlin Institute of Health, Institute of Hygiene and Environmental Medicine, Berlin, Germany; 2 National Reference Centre for Surveillance of Nosocomial Infections Charité University Medicine Berlin, Germany; 3 Potsdam Institute for Climate Impact Research, Potsdam, Germany; University of Maryland School of Medicine, UNITED STATES

## Abstract

**Background:**

The incidence of many infections is seasonal e.g. surgical site infections, urinary tract infection and bloodstream infections. We questioned whether there is seasonal variation even in climate-controlled hospitalized patients, and analyzed the influence of climate parameters on nosocomial bloodstream infections.

**Methods and findings:**

The retrospective cohort study is based on two databases: The German national surveillance system for nosocomial infections in intensive care units (ICU-KISS) from 2001 to 2015 and aggregated monthly climate data. Primary bloodstream infection (PBSI) is defined as a positive blood culture with one (or more) pathogen(s) which are not related to an infection on another site and which were not present at admission. Monthly infection data were matched with postal code, calendar month and corresponding monthly climate and weather data. All analyses were exploratory in nature. 1,196 ICUs reported data on PBSI to KISS. The ICUs were located in 779 hospitals and in 728 different postal codes in Germany. The majority of the 19,194 PBSI were caused by gram-positive bacteria. In total, the incidence density of BSI was 17% (IRR 1.168, 95%CI 1.076–1.268) higher in months with high temperatures (≥20°C) compared to months with low temperatures (<5°C). The effect was most prominent for gram-negatives; more than one third (38%) higher followed by gram-positives with 13%. Fungi reached their highest IRR at moderately warm temperatures between 15–20°C. At such temperatures fungi showed an increase of 33% compared to temperatures below 5°C. PBSI spiked in summer with a peak in July and August. PBSI differed by pathogen: The majority of bacteria increased with rising temperatures. Enterococci showed no seasonality. *S*. *pneumoniae* reached a peak in winter time. The association of the occurrence of PBSI and temperatures ≥20°C was stronger when the mean monthly temperature in the month prior to the occurrence of BSI was considered instead of the temperature in the month of the occurrence of BSI. High average temperatures ≥20°C increased the risk of the development of a PBSI by 16% compared with low temperatures <5°C.

**Conclusions:**

Most nosocomial infections are endogenous in nature; the microbiome plays a crucial role in host health. If gut and skin microbiome varies with season, environmental parameters will contribute to the observed incidence patterns. Similarly, the impact of global warming on both local weather patterns and extreme weather events may influence the acquisition of pathogens. A better understanding of the etiology of these infections is needed to provide guidance for future infection control strategies.

## Introduction

The incidence of many infections is seasonal [1]. Recognition of seasonal variations in the occurrence of infectious diseases dates back to the time of Hippocrates, who wrote in book III of the Aphorisms that “Every disease occurs at any season of the year but some of them more frequently occur and are of greater severity at certain times”. For Hippocrates, infections were influenced by environmental factors such as air, water, or food, and seasonal changes could give rise to diseases [2].

Recent studies revealed strong evidence that urinary tract infections peak in autumn [3]. Likewise, the risk of surgical site infections is also highly seasonal and is associated with warmer weather [4] [5]. Sepsis, one of the most life-threatening diseases, varies strikingly by season and by pathogen [6, 7]. Even with a relatively low incidence, bloodstream infections (BSI) generated a high burden of disease due to their high attributable mortality. A European point prevalence study on nosocomial infections estimated that more than 163,000 new cases of BSI occur every year in the EU [8].

Most studies revealing evidence of the seasonality of BSIs did not distinguish between hospital-onset and community-onset cases [9]. This might be of importance because different onsets may have different underlying foci of infection and, thus, a different epidemiology and natural history.

In this study we focus on the association between outside climate and weather parameters especially temperature with nosocomial infections. To answer the question whether there is outside temperature variation, even for patients in climate-controlled hospitals, we use comprehensive data of the national surveillance system for hospital infections in Germany (KISS) [10].

Worldwide we realized an increase in mean temperatures over the last decade’s across all seasons [11, 12]. A negative impact of high temperatures on the occurrence of BSI seems probable. This interaction plays an important role for infection prevention measures, especially with respect to the projections of climate researchers. An increase of 4°C in mean temperature until the end of this century in Germany is considered to be a probable scenario [13] in a world with little concern about the climate, so called business as usual.

## Objective

The aim of our exploratory study is to analyze the influence of climate parameters on nosocomial bloodstream infections and to examine whether different pathogens act in different ways.

## Methods

### Ethics statement

All infection data are anonymous and collected in accordance with the German recommendations for good epidemiological practice with respect to data protection [14]. A federal law, the German Protection Against Infection Act [15], regulates the prevention and management of infectious disease in humans. All hospitals are obliged to continually collect and analyze healthcare-associated infections and drug-resistant pathogens. This data is reported regularly to the National Reference Centre for the Surveillance for Nosocomial Infections. Ethical approval and informed consent were, therefore, not required.

### Data

The retrospective cohort study is based on two databases: ICU-KISS (2001–2015) for nosocomial infections in ICUs and aggregated monthly climate data of the “Deutscher Wetterdienst” (DWD).

### ICU-KISS

ICU-KISS is a module of the German hospital infection surveillance system “KISS” (Krankenhaus-Infektions-Surveillance-System), which collects data on nosocomial infections in ICUs.

The majority of the acute care hospitals in Germany participate voluntarily on a confidential basis [16, 17]. KISS collects the following nosocomial infections: primary bloodstream infections (PBSI), lower respiratory tract infections (LRTI) and urinary tract infections (UTI). The definitions of the infections are those of the Center for Disease Control and Prevention (CDC) [18]. We analyze in our study PBSI only. PBSI is defined as a positive blood culture with one (or more) pathogens which are not related to an infection on another site. For each infection, up to four pathogens can be recorded. Infections that occurred in ICUs are recorded on a case-basis. For a PBSI with skin germs, for example with coagulase negative Staphylococci (CNS), the definition also requires further clinical signs and the physician starts according to an antibiotic therapy. However, beginning in 2011 a new CDC definition of PBSI with skin germs was introduced. Since that time two separate blood cultures are required for skin germs in combination with other clinical signs. In addition, for each ICU the number of patients and patient days are recorded as denominator data.

### Climate data

Data from the climate station network of the German Meteorological Service (DWD) was used to analyze the seasonal cycle for different observed meteorological parameters: daily mean temperature, daily maximum temperature, daily precipitation, relative humidity, and the daily duration of sunshine [19]. Only long-term climate stations (n = 226) that provided data from 1961–2017 were included. Precipitation data was taken from 1297 long term measuring stations to satisfy the large spatial variability of rainfall events. The pre-selection was done analogously. First, daily meteorological data were interpolated on a 12 x 12 km grid [20]. With respect to temperature, the elevation of the destination and the five closest measuring points were considered. Next, the daily gridded data were aggregated to monthly climate indicators and assigned to postal codes coordinates using the nearest neighbor approach.

### Data collection

Monthly aggregated infection data were matched with postal code, calendar month, and monthly climate and weather data.

### Statistical analysis

The aim of this study was to analyze the association of BSI incidence density with different climate parameters, in particular with the ambient temperature, however, not with seasonality. The latitudes considered in this study, show pronounced seasonality, with summer months showing the highest temperatures and longest sunshine duration. Conversely, near the equator (near 0° altitude), seasonality plays only a marginal role, and temperatures on average are higher in contrast to altitudes further from the equator (for example in Germany 51° N). A publication by Fisman and coauthors revealed that higher temperatures are associated with higher incidence densities of nosocomial infections [21], but the higher temperatures near the equator showed no or minimal seasonality. Moreover, we conducted several analyses with different seasonal parameters, parametrized both as calendar months and as seasons (winter: December-February/ spring: Mars-May/ summer: June-August/ autumn: September-November). These additional analyses demonstrated a strong correlation between temperature and the selected seasonal parameters (data not shown). Analyses with these other seasonal parameters yielded similar results, as the analysis with a focus on temperature presented in this publication. Due to this correlation, we opted to include only the parameter temperature in our models, instead of a combination of temperature and other seasonal parameters.

The incidence rate of PBSI was defined as the number of PBSI per 10,000 patient days. The following weather and climate parameters were analyzed:

- mean daily temperature (°C) in the month of infection as a continuous parameter and mean daily temperature categorized in steps of 5 degrees Celsius (°C) steps (<5/ [5–10)/ [10–15)/ [15–20)/ ≥20) to investigate the proportionality (linearity) and /or non-linearity of association.
- vapor pressure (hPa), relative humidity (%), duration of sunshine (h), amount of precipitation (mm), number days with heavy rain (>20mm), heat days (number of days with maximum temperatures >25°C), ice days (number of days with maximum temperatures <0°C).

In addition, we analyzed the association of temperature parameters with a temporal shift/delay of 1, 2 and 3 months prior to infection to reflect endogenous colonization and other effects.

Incidence rates with 95% confidence intervals were calculated for the outcome all PBSI, PBSI with gram-positive or gram-negative pathogens or fungi and for PBSI with several pathogens (N>100).

In the multivariable analysis, for all outcome above and for each climate and weather parameter adjusted incidence rate ratio (IRR) with the 95% confidence interval were calculated for all outcomes above and for each climate and weather parameter. For this, time series analyses using generalized linear models were performed to estimate the association of the number of PBSI per month with weather and climate parameters and further confounders. Because, due to the management, observations within an ICU are not statistically independent, adjusted incidence rate ratios (IRR) with 95% confidence intervals (CI) were estimated. They were based on generalized estimating equation (GEE) models which account for this clustering effect by using an exchangeable correlation structure [22]. We used Poisson distribution in the models and the log number of patient days during each month was treated as an offset in the model.

The following parameters were considered as confounders concerning the occurrence of PBSI: length of stay (LOS, days) and device use (device days per 100 patient days) for central venous catheter days, urinary tract catheter days, and ventilator days to adjust for the severity of illness; and, long-term trends between 2000 and 2016 (linear, quadratic and cubic). The following structural parameters were used: type of ICU (medical, surgical, interdisciplinary, other), size of ICU (</≥12 beds), type of hospital in which ICU is located (university, tertiary / other) and size of hospital in which ICU is located (</≥600 beds). The multivariable model building strategy was performed in two steps for each pathogen.

1. All confounding parameters were included in a full GEE model with the outcome (number) all PBSI, and non-significant parameters were excluded stepwise. The selection criterion was the smallest Chi-square value and p> = 0.05 in the type III score statistic.
2. For all PBSI and PBSI with pathogen groups and with several pathogens, a GEE model was calculated with the significant confounding parameters from the first step and the temperature and weather parameters. Temperature was analyzed as continuous and categorical parameter all other weather parameters were analyzed as a continuous variable in the model.

The quasi-likelihood information criterion as a modification of the Akaike information criterion was used as goodness-of-fit measure in the GEE model. P-values less than 0.05 were considered significant. Analyses were exploratory in nature. All analyses were performed using SPSS [IBM SPSS statistics, Somer, NY, USA] and SAS [SAS Institute, Cary, NC, USA].

## Results

1,196 ICUs reported data on primary bloodstream infections to the German National surveillance system KISS between 2001 and 2015. The ICUs were located in 779 hospitals and in 728 different postal codes in Germany. The majority of the 19,194 PBSI were caused by gram-positive bacteria. Table 1 shows the basic characteristics of the 1,196 ICUs.

**Table 1:** Basic characteristics of 1,169 ICUs participating in KISS (German hospital infection surveillance system), 2001–2015.

Study unit data (1,169 ICUs)	N (%) or Median (IQR)
Total	1,169 (100%)
Type of ICU	
Interdisciplinary	665 (55.6%)
Medical	180 (15.1%)
Surgical	175 (14.6%)
Other	176 (14.7%)
ICU Size (beds), Median (IQR)	12 (8–15)
Type of Hospital	
University or Tertiary	562 (47%)
Other than university or tertiary	634 (53%)
Hospital size (beds), Median (IQR)	416 (241.5–735)
Participation month, Median (IQR)	55 (24–101)
Patients, patient days, device days	N
Patients	6,590,252
Patient days	23,896,847
Urinary cather days	19,109,132
Invasive ventilation days	9,284,565
Central venous catheter days	15,758,589
Length of stay, Device use (per 100 patient days)	Median (IQR)
Length of stay (days)	3.62 (2.78–4.96)
Central venuous catheter use	62.6 (47.2–77.3)
Urinary tract catheter use	81.49 (70.42–89.97)
Invasive ventilation use	33.28 (22.79–46.23)
Primary bloodstream infection (PBSI)	N; incidence rate per 10,000 patient days (95% CI)
all PBSI	19,194; 8.03 (7.92–8.15)
PBSI with gram-positive bacteria	12,831; 5.37 (5.28–5.46)
Coagulase negative staphylococci	6,525; 2.73 (2.66–2.8)
Enterococcus spp.	3,654; 1.53 (1.48–1.58)
Staphylococcus aureus	2,954; 1.24 (1.19–1.28)
Streptococcus spp.	148; 0.06 (0.05–0.07)
Corynebacteriaceae spp.	115; 0.05 (0.04–0.06)
PBSI with gram-negative bacteria	4,550; 1.9 (1.85–1.96)
Escherichia coli	1,006; 0.42 (0.4–0.45)
Klebsiella spp.	1,005; 0.42 (0.39–0.45)
Enterobacter spp.	820; 0.34 (0.32–0.37)
Pseudomonas aeruginosa	689; 0.29 (0.27–0.31)
Serratia spp.	335; 0.14 (0.13–0.16)
Acinetobacter spp.	219; 0.09 (0.08–0.1)
Proteus spp.	197; 0.08 (0.07–0.09)
Other enterobacteriaceae	135; 0.06 (0.05–0.07)
Stenotrophomonas maltophilia	134; 0.06 (0.05–0.07)
Citrobacter spp.	119; 0.05 (0.04–0.06)
Bacteroides spp.	102; 0.04 (0.03–0.05)
PBSI with fungi	1,543; 0.65 (0.61–0.68)
Candida albicans	1,062; 0.44 (0.42–0.47)
Non-albicans Candida	332; 0.14 (0.12–0.15)
Other fungi than Candida spp.	174; 0.07 (0.06–0.08)

N, number; 95%CI, 95% confidence interval; IQR, interquartile range; PBSI, primary bloodstream infection; CVC, central venous catheter.

**Fig 1:**
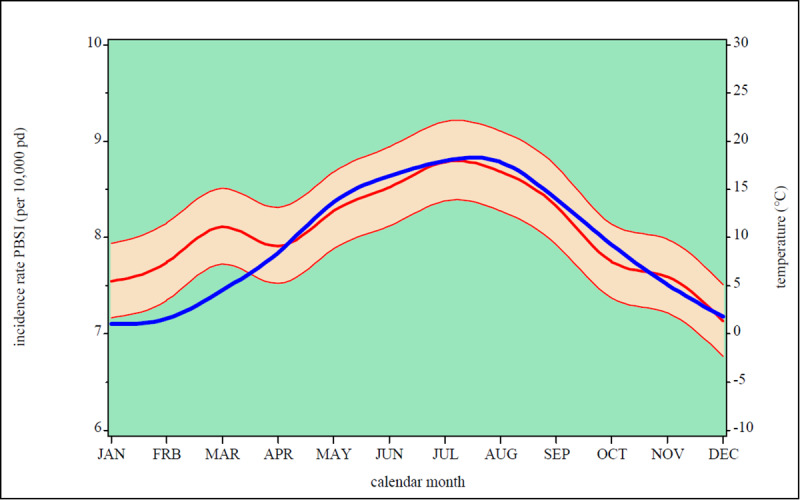
Incidence rate primary bloodstream infection (per 10,000 patient days, bold red line) with 95% confidence interval (red area) and mean temperature in degrees Celsius (bold blue line) stratified by calendar month.

The seasonal variability of PBSI for our ICUs is depicted in Fig 1. An increase in mean daily temperature accompanies an increase in the occurrence of PBSI. The incidence rate for our outcome all PBSI, PBSI with gram-positive or gram-negative pathogens or fungi and for PBSI with several pathogens stratified by steps of 5 degrees Celsius of the mean daily temperature showed S1 Table.

**Fig 2:**
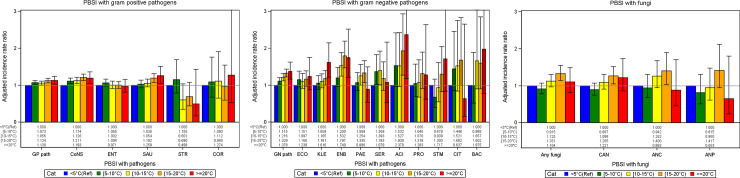
**a.** Adjusted incidence rate ratio (IRR) for primary bloodstream infections (PBSI) with gram-positive pathogens for the temperature in the month of PBSI compared to temperatures less 5°C. Legend a. GP path, gram positive pathogens; CoNS, coagulase negative staphylococci; ENT, Enterococci; SAU, Staphyloccus aureus; STR, Streptococcus pneumoniae; COR, Corynebacterium. **b.** Adjusted incidence rate ratio (IRR) for primary bloodstream infections (PBSI) with gram-negative pathogens for the temperature in the month of PBSI compared to temperatures less 5°C. Legend b. GN path, gram-negative; ECO, E.coli; KLE, Klebsiella spp.; ENB, Enterobacter spp.; PAE, Pseudomonas aeruginosa; SER, Serratia spp.; ACI, Acinetobacter ssp.; PRO, Proteus spp.; STM, Stenothropomonas maltophila; CIT, Citrobacter spp.; BAC, Bacteroides spp. **c.** Adjusted incidence rate ratio (IRR) for primary bloodstream infections (PBSI) with fungi for the temperature in the month of PBSI compared to temperatures less 5°C. Legend c. Any fungi, fungi; CAN, Candiada albicans; ANC, other than albicans Candida; ANP, other fungi than Candida.

The adjusted incidence rate ratios (IRR) for primary bloodstream infections with pathogens or group of pathogens for the temperature in the month of occurrence of BSI are shown in Fig 2A–2C and S2 Table. The multivariable analysis were performed using generalized estimating equation (GEE) models which are adjusted for length of stay, central venous catheter use, invasive ventilation, type of ICU, size of hospital, long-term trends and accounted for clustering.

In total, the incidence rate of BSI was 17% (IRR 1.169, 95%CI (1.077–1.269)) higher in months with high temperatures (≥20°C) when compared to months with low temperatures (<5°C) (Table 2). The effect was most prominent for gram-negatives with more than one third (38%) higher (Fig 2B, S2 Table) followed by gram-positives with 13% (Fig 2A, S2 Table). The exception is *S*. *pneumoniae* which behaves differently from other gram-positives. In *S*. *pneumoniae*, the IRR was 0.498 (95%CI 0.174–1.429) for months ≥ 20°C compared to months <5°C. In other words, *S*. *pneumoniae* occurred 50% less frequently at such high temperatures than at low temperatures. Fungi achieve their highest IRR at moderately warm temperatures between 15–20°C. At those temperatures fungi showed an increase of 33% compared to temperatures below 5°C (IRR = 1.331 (95%CI 1.144–1.549) (Fig 2C, S2 Table).

**Table 2:** Adjusted incidence rate ratios (IRR) for the outcome all primary bloodstream infections (PBSI) with the temperature in the month of PBSI compared to temperatures less 5°C.

Parameter	Category	IRR (95%CI)	p-value
Temperature (°C)	<5°C	1 = reference	
	[5–10°C)	1.054 (1.014–1.096)	0.0082
	[10–15°C)	1.084 (1.040–1.130)	0.0001
	[15–20°C)	1.170 (1.122–1.221)	< .0001
	> = 20°C	1.169 (1.077–1.269)	0.0002
Central venous catheter use	per 1 CVC day/100pd	1.010 (1.007–1.012)	< .0001
Invasive ventilation	per 1 INV day/100pd	1.010 (1.007–1.012)	< .0001
Length of stay	per 1 day	1.005 (1.001–1.009)	0.0172
Type of ICU	Interdisciplinary	1 = reference	
	Medical	1.076 (0.87–1.331)	0.4977
	Surgical	0.910 (0.758–1.093)	0.3122
	Other	1.257 (1.015–1.557)	0.0361
Hospital size	<600 beds	1 = reference	
	> = 600 beds	1.257 (1.059–1.492)	0.009
Long-term trends	Quadratic	0.993 (0.991–0.996)	< .0001
Long-term trends	Cubic	1.0003 (1.0001–1.0005)	0.0004

IRR, incidene rate ratio; CI, confidence interval; CVC, central venous catheter; INV, invasive ventilation.

**Fig 3:**
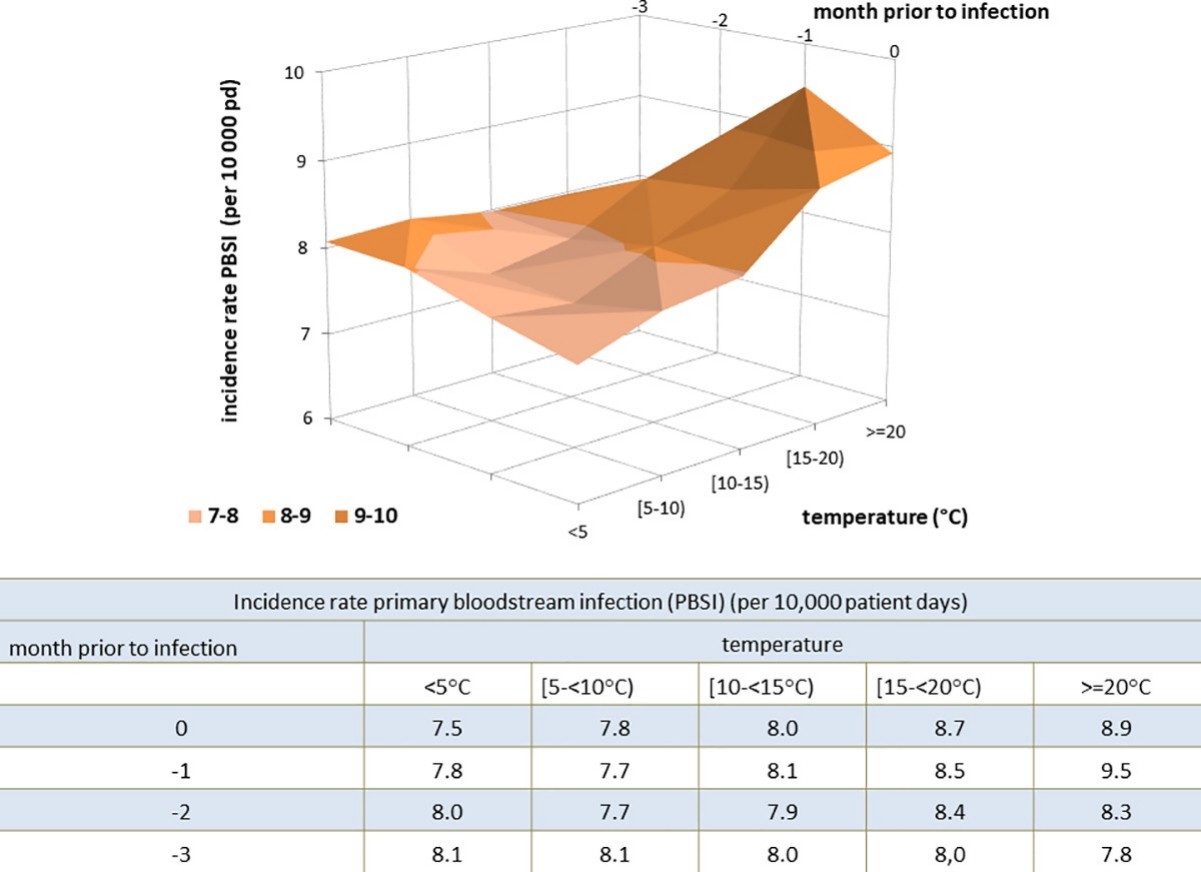
Incidence rate of all primary bloodstream infections PBSI per 10,000 patient days depending on temperature in the month of infection and one, two and three-month (lag) prior to the infection. Incidence rate is highest (9.5 PBSI per 10,000 patient days) in a month, if the temperature in the month prior was greater than 20°C.

To account for the temporal shift between the temperature and the frequency of pathogens associated to the BSI, we considered the temperature in the actual month and for a one, two, and three month lag prior to the occurrence of the pathogen associated to the BSI (Fig 3). The incidence rate of all PBSI over the 15-year period corresponded most highly at temperatures above 20°C with the mean temperature one month before the onset of infection.

The statistically significant results of multivariate regression analysis for the outcome all PBSI are shown in Table 2. The two parameters with the highest impact were warmer temperatures ≥ 20°C (compared to temperatures less 5°C) and hospitals with more than 600 beds.

Multivariable analysis were performed using generalized estimating equation (GEE) models: parameters considered in the initial GEE model were central venous catheter use, invasive ventilation, urinary tract catheter use, length of stay, type of ICU (interdisciplinary/ medical/ surgical/ other), size of ICU (</ ≥12 beds), size of hospital (</ ≥600 beds), bed occupancy, type of hospital (university or tertiary/ other), and long-term trends linear, quadratic and cubic. Non-significant parameters were excluded by variable selection stepwise backward.

The adjusted incidence rate ratios for PBSI for various meteorological parameters revealed that not only high temperatures correlate significantly, vapor pressure and inversely relative humidity do too (Table 3). Primary bloodstream infections caused by bacteria or fungi are the higher the warmer and relatively drier the weather is. This is especially the case for gram-negatives.

**Table 3:** Adjusted incidence rate ratios (IRR) for all primary bloodstream infections (PBSI), for PBSI with gram-positive, for PBSI with gram-negative and for PBSI with fungi with different meteorological parameters. Table showed the IRR, 95% confidence interval and p-values of the multivariable GEE models adjusted for length of stay, central venous catheter use, invasive ventilation, type of ICU, size of hospital, long-term trend and accounted for clustering

Pathogen (group)	Longitude (per 1°)	Latitude (per 1°)	Rainfall (ml/m^2^)	Relative humidity (%)	Sun time (h)	Heat days (>20°C)	Ice days (<0°C)	Days with heavy rain (>20mm)	Vapor pressure (hPa)	Mean daily temp (°C)
**All PBSI**	1.006 (0.977–1.036)	**1.052** (1.007–1.099)	1 (1–1.001)	**0.994** (0.992–0.996)	**1.001** (1–1.001)	**1.017** (1.012–1.024)	**0.992** (0.987–0.996)	1.015 (0.992–1.038)	**1.016** (1.012–1.021)	**1.009** (1.007–1.012)
	p = 0.676	p = 0.022	p = 0.373	p≤0.001	p≤0.001	p≤0.001	p≤0.001	p = 0.214	p≤0.001	p≤0.001≤
**PBSI with gram-positive bacteria**	1.001 (0.969–1.034)	**1.072** (1.015–1.131)	1 (1–1.001)	**0.994** (0.991–0.996)	**1.001** (1–1.001)	**1.013** (1.006–1.02)	**0.993** (0.988–0.999)	1.007 (0.980–1.035)	**1.009** (1.003–1.015)	**1.007** (1.004–1.010)
	p = 0.937	p = 0.012	p = 0.561	p≤0.001	p≤0.001	p≤0.001	p = 0.025	p = 0.604	p = 0.002	p≤0.001
**PBSI with gram-negative bacteria**	1.005 (0.971–1.04)	1.031 (0.991–1.072)	1 (0.999–1.001)	**0.995** (0.991–0.998)	**1.001** (1.001–1.001)	**1.031** (1.019–1.043)	**0.981** (0.971–0.991)	1.018 (0.976–1.062)	**1.036** (1.028–1.045)	**1.018** (1.013–1.023)
	p = 0.780	p = 0.132	p = 0.723	p = 0.004	p≤0.001	p≤0.001	p≤0.001	p = 0.397	p≤0.001	p≤0.001
**PBSI with fungi**	1.014 (0.976–1.054)	1.022 (0.974–1.073)	1.001 (0.999–1.002)	**0.990** (0.984–0.997)	**1.001** (1.001–1.002)	**1.027** (1.006–1.049)	**0.980** (0.964–0.997)	1.068 (0.999–1.142)	**1.037** (1.02–1.054)	**1.020** (1.011–1.029)
	p = 0.463	p = 0.379	p = 0.296	p = 0.003	p≤0.001	p = 0.010	p = 0.024	p = 0.055	p≤0.001	p≤0.001

**Bold**, if statistically significant p<0.05

vapor pressure (hPa), relative humidity (%), duration of sunshine (h), amount of precipitation (mm), number days with heavy rain (>20mm), heat days (number of days with maximum temperatures >25°C), ice days (number of days with maximum temperatures <0°C).

## Conclusion

The six main findings of this retrospective and explorative cohort study of more than 6.5 million ICU patients and on more than 19,000 BSI in a 15-year period are as follows:

1. Even nosocomial BSI are associated to outside temperature and spike in months with highest temperatures.
2. This is especially true for gram negatives but also–to a lesser extent–for gram positives and fungi.
3. Pathogens differ. The majority of bacteria increase with rising temperatures. Enterococci, however, show no seasonality. S. pneumoniae peaks in months with lowest temperatures. BSI due to fungi, P. aeruginosa and Serratia spp. peaked at moderately warm temperatures between 15 and 20°C.
4. The association of the occurrence of BSI and temperatures >20°C was stronger when the mean monthly temperature in the month prior to the occurrence of BSI was considered instead of the temperature in the month of occurrence.
5. Known risk factors for BSI are central venous catheters, invasive ventilation and length of stay. Environmental factors seem also to contribute: high average temperatures >20°C increase the risk for the development of a BSI by 16% compared with low temperatures <5°C.
6. Contrary to our expectations that warm and humid weather would have an impact on BSI, it was warm and relatively dry weather with which it was associated.

In Table 4 we have summarized relevant studies on the effect of meteorological factors on gram-positive and gram-negative bloodstream infections. The majority of the studies show that gram-negative bloodstream infections peak in warmer months. Results for gram-positives are inconsistent, except for *S*. *pneumoniae* BSI, which peaks in winter time.

**Table 4:** Relevant and related studies on the effect of meteorological factors on bloodstream infection (BSI).

Author	Sites/study period/ number of BSI	Relevant findings	Impact of higher temperature on gram negative BSI	Impact of higher temperature on gram positive BSI
**BSI all pathogens**				
Eber (2011) [6]	132 US hospitals 1999–2006 211,697 BSI	BSIs due to Gram negatives were more frequent in summer relative to winter (12.2% for E. coli and 51.8% for Acinetobacter). No summer peak for BSI due to Gram positives	yes	No for Entero-coccus
Alcorn (2013) [23]	1 Australian hospital 2001–2011 440 BSI	significant increase in the frequency of GN BSI was observed in nonhospitalized patients during the summer months (P = .03) but not in climate-controlled hospitalized patients	yes	no
Gonçalves-Pereira (2013) [24]	17 Portuguese ICUs 2004–2005 160 BSI	gram-negative community acquired BSI were more common in the summer, whereas in the winter, gram-positive infections were more frequent	yes	
Fisman (2014) [21]	23 international medical centers 2007–2011 64,739 BSI	the overall P-value for seasonal oscillation was significant	yes	
Caldeira (2015) [25]	1 Brazilian hospital 2005–2010 1,619 HA-BSI	temperature was positively associated with the recovery of gram-negative bacilli or Acinetobacter baumannii	yes	
**BSI due to specific pathogen**				
Anderson (2008) [26]	4 hospitals 2001–2006 1,189 K. pneumoniae BSI	BSI due to K. pneumoniae higher in the 4 warmest months	yes for *K*. *pneumoniae*	
Tvedebrink (2008) [27]	1 Danish county 1995–2002 714 S. pneumoniae BSI	distinctive seasonal variation in the incidence of pneumococcal bacteraemia in Denmark, with summer troughs and winter peaks		yes for *S*. *pneumoniae*
Al-Hasan (2009) [28]	2 US medical centers 1998–2007 461 E.coli BSI	35% increase of E. coli BSI during the warmest four months; 7% increase for each 5.5°C increase in average temperature	yes for *E*.*coli*	
Al-Hasan (2010) [29]	2 US medical centers 1998–2007 127 Klebsiella BSI	no seasonal variation in incidence rate of *Klebsiella spp* BSI	no for *Klebsiella spp*.	
Chazan (2011) [30]	1 Israeli hospital 2001–2008 2,810 BSI	significantly higher rates of E. coli BSI in the summer compared to transitional and winter seasons	yes for *E*.*coli*	
Kato (2014) [31]	1 Japanese hospital 2008 to 2013 51 B. cereus BSI	significantly more from June to September than from January to April		yes for *B*. *cereus*
Deeny (2015) [32]	167 UK NHS trusts 2011–2013 79,155 E. coli BSIs	for the community-onset cases statistically significant seasonal variation over time nationally; but not in hospital-onset cases	yes for community acquired *E*. *coli*	
Gradel (2016) [9]	3 Danish Health regions 2000–2011 16,006 E. coli, 6,924 S. aureus, and 4,884 S. pneumoniae BSI in total 27,808 BSI	For E. coli, the seasonal variation was highest for community-acquired cases and was missing for hospital-acquired cases. No seasonal variation for S. aureus. S. pneumoniae showed high seasonal variation	yes for community acquired *E*. *coli*	no for *S*. *aureus* yes for *S*. *pneumoniae*

Highlighed are the four studies with more than 27.000 BSI included

An obvious question is whether and how the climatic factors influence the occurrence of pathogens and of nosocomial infections like sepsis. Most nosocomial infections are endogenous in nature. Under basal conditions, the microbiome plays a crucial role in host health. Several changes occur to gut physiology in septic patients, due either to extrinsic factors (antibiotics and parenteral nutrition) or intrinsic factors (systemic inflammation and gut leakage). These changes, in turn, influence the composition of the enteric flora [33, 34]. Imbalances between pathogenic and symbiotic commensal species can be seen even before the onset of disease and are also influenced by environmental factors [35].

BSIs in the healthcare setting are classified as primary BSI, related to a central venous catheter or other hospital-acquired source, or secondary BSI, such as those associated with abscess or pneumonia. It has become evident that some BSIs that occur in patients with CVCs do not arise from the catheter but instead are derived from other sources, such as translocation of bacteria through non-intact mucosa [36, 37]. Bacterial translocation is the invasion of indigenous intestinal bacteria through the gut mucosa to normally sterile tissues such as the mesenteric lymph nodes and the internal organs. It includes passage of antigens or endotoxins from the gut lumen into the circulation causing systemic inflammation. The Centers for Disease Control and Prevention developed a modification of the CLABSI definition, termed mucosal barrier injury laboratory-confirmed BSI [38]. It can be hypothesized that this pathophysiology plays not only a role in hematology and oncology patients but also in ICU patients.

The human microbiome varies by season [39]. There has been speculation about the environmental changes that occur in warmer months for both the number and types of organisms found in the environment, and also regarding colonization of human skin. The in vitro optimal growth temperatures for several gram-negative organisms falls between 32°C and 36°C, making it intuitive to expect higher amounts of these organisms in the environment during summer [23].

We know even less—compared to bacterial communities—about the human gut mycobiome. The mycobiome is low in diversity and dominated by yeast including Saccharomyces, Malassezia, and Candida. But, there is evidence that a core gut mycobiome may exist [40]. Although, it is unclear whether the apparent rise in the incidence of fungal BSI at moderately warm temperatures is directly related to exposure of the human host to warmer environmental conditions or whether it is some sort of epiphenomenon.

Our study focused on nosocomial primary BSI. The results of our study are only partly in line with the findings of other four studies (each including more than 27,000 BSIs).

The largest study done thus far was in the US on more than 211,000 BSIs, including both community and hospital-acquired [6]. Eber et al. concluded that a higher outdoor temperature was consistently associated with an increased frequency of BSIs caused by gram-negative bacteria (*Acinetobacter*, *E*. *coli*, *K*. *pneumonia*, and *P*. *aeruginosa*). Increased temperature was associated with a relatively modest but statistically significant increase in *S*. *aureus* BSI frequency but no significant change in *Enterococcus* BSI frequency. For most organisms analyzed, neither monthly precipitation levels nor humidity levels were associated significantly with an increased frequency of BSIs in multivariate analyses. These results are essentially what we found in nosocomial BSI. Eber *et al* discussed the fact that higher temperatures facilitate increased growth of bacteria in the environment, which may, in turn, increase colonization of humans and that elevated temperatures may be associated with increased virulence of gram-negative bacteria.

Fisman *et al*. looked within a network of 23 international centers for geographical and climatic factors associated with this variability of BSI: for bacteremic patients. The likelihood that the infection was due to gram-negative bacteria was significantly greater in locations closest to the equator and during warmer months of the year [21]. In our study, we did not see a difference for BSI with respect to latitude (48°N-55°N) but very likely Germany is too small to confirm differences.

The results of our study are in contrast to the UK national trust study by Deeny *et al*. [32]. They showed no seasonality in hospital-onset *E*. *coli* BSIs, which constitute 25% of their full dataset. Furthermore, the weekly impact of seasonality *E*. *coli* BSI was statistically significant only in the North of England but not in London or the rest of England. Likewise, a Danish study reported that the seasonal variation was highest for community-acquired cases of *E*.*coli* BSI, that is was lower for health-care associated cases, and it was not present for hospital-acquired cases [9]. No seasonal variation was observed for *S*. *aureus* except for some locations of infection (intravascular catheters and unknown). *S*. *pneumoniae* showed high seasonal variation. We believe that methodological differences alone, such as the mode of data collection, cannot explain the differences.

The strengths of our study are the multicenter design with 1196 ICUs reporting data on primary bloodstream infections by an active surveillance system, the relatively long study period of 15 years, and that all isolates were considered, i.e. not only bacteria but also fungi in blood cultures. We generated sound results by adjusting risk factors and by associating climate data with BSI data by postal codes. This association with meteorological factors was far more detailed than in most other studies. Our study has also limitations. First, all data are reported voluntarily which implies heterogeneity of surveillance. Second, conclusions from our study cannot be generalized for other settings, e.g. neonates, because we analyzed only adult ICU patients. Third, we assigned the postal codes of the hospital with the corresponding climate data. This postal code does not necessarily represent where patient was living before admission to the hospital. If climate has an impact on the microbiome and microbiome at temperatures >20°C one month before onset of infection this parameter might be not precise. Fourth, our analysis considered only ICU acquired primary BSI. They are not included BSI within the first three days on ICU and secondary BSI.

The knowledge that BSIs were on average 17 percent more likely to occur in summer compared with winter is of importance. Studies should consider temperature as additional parameter. For example, studies–even randomized controlled trials–analyzing infections caused by gram-negatives in summer versus winter could considerably bias results if they do not control for this parameter.

Future studies of factors, such as host factors and environmental variables that may be contributing to these observed incidence patterns are needed. Similarly, the impact of global warming on both local weather patterns and extreme weather events may impact the acquisition of pathogens. These findings may then contribute to our understanding of the etiology of these infections and may provide guidance for future infection control strategies.

## Supporting information

S1 Table Incidence rate (per 10,000 patient days) for the outcome all primary bloodstream infection (PBSI), PBSI with gram-positive or gram-negative pathogens or fungi and for PBSI with several pathogens stratified by steps of 5 degrees Celsius of the mean daily temperature. Data of 1,169 ICUs participating in KISS (German hospital infection surveillance system), 2001–2015.
(DOCX)

S2 Table Adjusted Incidence rate ratio (IRR) with 95% confidence interval (CI) for the outcome all primary bloodstream infection (PBSI), PBSI with gram-positive or gram-negative pathogens or fungi and for PBSI with several pathogens with the mean daily temperature in the month of PBSI compared to temperatures less 5°C. Data of 1,169 ICUs participating in KISS (German hospital infection surveillance system), 2001–2015.
(DOCX)
