# Supplementary material for: Summer, sun and sepsis—The influence of outside temperature on nosocomial bloodstream infections: A cohort study and review of the literature
Source: PLoS One. 2020 Jun 19;15(6):e0234656. doi: 10.1371/journal.pone.0234656 (PMC7304998; doi:10.1371/journal.pone.0234656)
Supplement: S1 Table — Data of 1,169 ICUs participating in KISS (German hospital infection surveillance system), 2001–2015. (DOCX) [file pone.0234656.s001.docx]

**Table S1**: Incidence rate (per 10,000 patient days) for the outcome all primary bloodstream infection (PBSI), PBSI with gram-positive or gram-negative pathogens or fungi and for PBSI with several pathogens stratified by steps of 5 degrees Celsius of the mean daily temperature. Data of 1,169 ICUs participating in KISS (German hospital infection surveillance system), 2001-2015.

| Parameter | total | temperature <5°C | [5-10°C) | [10-15°C) | [15-20°C) | >=20°C |
| --- | --- | --- | --- | --- | --- | --- |
| Patients, Patient and Device days (number) | | | | | | |
| patients | 6,590,252 | 1,796,929 | 1,589,415 | 1,289,076 | 1,692,626 | 222,206 |
| patient days | 23,896,847 | 6,628,800 | 5,753,410 | 4,622,830 | 6,071,304 | 820,503 |
| UTC days | 19,109,132 | 5,333,027 | 4,585,786 | 3,678,742 | 4,851,061 | 660,516 |
| invasive-ventilation days | 9,284,565 | 2,651,099 | 2,233,102 | 1,760,070 | 2,317,490 | 322,804 |
| CVC days | 15,758,589 | 4,403,619 | 3,773,277 | 3,020,737 | 4,002,712 | 558,244 |
| primary bloodstream infection (PBSI), number, incidence density per 10,000 patient days (95%CI) | | | | | | |
| all PBSI | 19194, 8.03 (7.92-8.15) | 4977, 7.51 (7.3-7.72) | 4506, 7.83 (7.6-8.06) | 3681, 7.96 (7.71-8.22) | 5298, 8.73 (8.49-8.96) | 732, 8.92 (8.29-9.59) |
| PBSI with gram-positive bacteria | 12831, 5.37 (5.28-5.46) | 3367, 5.08 (4.91-5.25) | 3102, 5.39 (5.2-5.58) | 2443, 5.28 (5.08-5.5) | 3443, 5.67 (5.48-5.86) | 476, 5.8 (5.29-6.35) |
| CoNS | 6525, 2.73 (2.66-2.8) | 1641, 2.48 (2.36-2.6) | 1559, 2.71 (2.58-2.85) | 1281, 2.77 (2.62-2.93) | 1806, 2.97 (2.84-3.12) | 238, 2.9 (2.54-3.29) |
| Enterococcus spp. | 3654, 1.53 (1.48-1.58) | 1011, 1.53 (1.43-1.62) | 931, 1.62 (1.52-1.73) | 676, 1.46 (1.35-1.58) | 909, 1.50 (1.40-1.60) | 127, 1.55 (1.29-1.84) |
| S.aureus | 2954, 1.24 (1.19-1.28) | 764, 1.15 (1.07-1.24) | 673, 1.17 (1.08-1.26) | 561, 1.21 (1.12-1.32) | 836, 1.38 (1.29-1.47) | 120, 1.46 (1.21-1.75) |
| Streptococcus spp. | 148, 0.06 (0.05-0.07) | 49, 0.07 (0.05-0.10) | 46, 0.08 (0.06-0.11) | 20, 0.04 (0.03-0.07) | 30, 0.05 (0.03-0.07) | 3, 0.04 (0.01-0.11) |
| Corynebacteriaceae spp. | 115, 0.05 (0.04-0.06) | 31, 0.05 (0.03-0.07) | 28, 0.05 (0.03-0.07) | 24, 0.05 (0.03-0.08) | 27, 0.04 (0.03-0.06) | 5, 0.06 (0.02-0.14) |
| PBSI with gram-negative bacteria | 4550, 1.9 (1.85-1.96) | 1096, 1.65 (1.56-1.75) | 1040, 1.81 (1.70-1.92) | 902, 1.95 (1.83-2.08) | 1320, 2.17 (2.06-2.29) | 192, 2.34 (2.02-2.7) |
| E. coli | 1006, 0.42 (0.40-0.45) | 254, 0.38 (0.34-0.43) | 254, 0.44 (0.39-0.50) | 189, 0.41 (0.35-0.47) | 269, 0.44 (0.39-0.5) | 40, 0.49 (0.35-0.66) |
| Klebsiella spp. | 1005, 0.42 (0.39-0.45) | 257, 0.39 (0.34-0.44) | 230, 0.40 (0.35-0.45) | 191, 0.41 (0.36-0.48) | 274, 0.45 (0.4-0.51) | 53, 0.65 (0.48-0.84) |
| Enterobacter spp. | 820, 0.34 (0.32-0.37) | 169, 0.25 (0.22-0.3) | 169, 0.29 (0.25-0.34) | 173, 0.37 (0.32-0.43) | 271, 0.45 (0.39-0.5) | 38, 0.46 (0.33-0.64) |
| P. aeruginosa | 689, 0.29 (0.27-0.31) | 171, 0.26 (0.22-0.3) | 150, 0.26 (0.22-0.31) | 145, 0.31 (0.26-0.37) | 204, 0.34 (0.29-0.39) | 19, 0.23 (0.14-0.36) |
| Serratia spp. | 335, 0.14 (0.13-0.16) | 78, 0.12 (0.09-0.15) | 90, 0.16 (0.13-0.19) | 73, 0.16 (0.12-0.20) | 83, 0.14 (0.11-0.17) | 11, 0.13 (0.07-0.24) |
| Acinetobacter spp. | 219, 0.09 (0.08-0.10) | 41, 0.06 (0.04-0.08) | 52, 0.09 (0.07-0.12) | 43, 0.09 (0.07-0.13) | 71, 0.12 (0.09-0.15) | 12, 0.15 (0.08-0.26) |
| Proteus spp. | 197, 0.08 (0.07-0.09) | 50, 0.08 (0.06-0.10) | 44, 0.08 (0.06-0.10) | 36, 0.08 (0.05-0.11) | 59, 0.1 (0.07-0.13) | 8, 0.10 (0.04-0.19) |
| S.maltophilia | 134, 0.06 (0.05-0.07) | 37, 0.06 (0.04-0.08) | 21, 0.04 (0.02-0.06) | 24, 0.05 (0.03-0.08) | 44, 0.07 (0.05-0.1) | 8, 0.10 (0.04-0.19) |
| Citrobacter spp. | 119, 0.05 (0.04-0.06) | 25, 0.04 (0.02-0.06) | 30, 0.05 (0.04-0.07) | 25, 0.05 (0.03-0.08) | 37, 0.06 (0.04-0.08) | 2, 0.02 (0-0.09) |
| Bacteroides spp. | 102, 0.04 (0.03-0.05) | 22, 0.03 (0.02-0.05) | 18, 0.03 (0.02-0.05) | 24, 0.05 (0.03-0.08) | 32, 0.05 (0.04-0.07) | 6, 0.07 (0.03-0.16) |
| PBSI with fungi | 1543, 0.65 (0.61-0.68) | 397, 0.6 (0.54-0.66) | 313, 0.54 (0.49-0.61) | 301, 0.65 (0.58-0.73) | 477, 0.79 (0.72-0.86) | 55, 0.67 (0.5-0.87) |
| C.albicans | 1062, 0.44 (0.42-0.47) | 280, 0.42 (0.37-0.47) | 215, 0.37 (0.33-0.43) | 205, 0.44 (0.38-0.51) | 319, 0.53 (0.47-0.59) | 43, 0.52 (0.38-0.71) |
| other than albicans Candida | 332, 0.14 (0.12-0.15) | 82, 0.12 (0.1-0.15) | 67, 0.12 (0.09-0.15) | 71, 0.15 (0.12-0.19) | 103, 0.17 (0.14-0.21) | 9, 0.11 (0.05-0.21) |
| other fungi | 174, 0.07 (0.06-0.08) | 47, 0.07 (0.05-0.09) | 33, 0.06 (0.04-0.08) | 30, 0.06 (0.04-0.09) | 60, 0.1 (0.08-0.13) | 4, 0.05 (0.01-0.12) |

PBSI, primary bloodstream infection; CoNS, coagulase negative staphylococci.
