# Supplementary material for: Summer, sun and sepsis—The influence of outside temperature on nosocomial bloodstream infections: A cohort study and review of the literature
Source: PLoS One. 2020 Jun 19;15(6):e0234656. doi: 10.1371/journal.pone.0234656 (PMC7304998; doi:10.1371/journal.pone.0234656)
Supplement: S2 Table — Data of 1,169 ICUs participating in KISS (German hospital infection surveillance system), 2001–2015. (DOCX) [file pone.0234656.s002.docx]

**Table S2**: Adjusted Incidence rate ratio (IRR) with 95% confidence interval (CI) for the outcome all primary bloodstream infection (PBSI), PBSI with gram-positive or gram-negative pathogens or fungi and for PBSI with several pathogens with the mean daily temperature in the month of PBSI compared to temperatures less 5°C. Data of 1,169 ICUs participating in KISS (German hospital infection surveillance system), 2001-2015.

|  | Model with continuous temperature parameter | Modell with categorized temperature parameter | | | |
| --- | --- | --- | --- | --- | --- |
| outcome | mean daily temperature | Mean daily temperature interval (°C) compared to <5°C | | | |
| PBSI with Pathogen (group) | per 1°C | [5-10°C) | [10-15°C) | [15-20°C) | >=20°C |
| all PBSI | 1.009 (1.007-1.012) p<=0.001 | 1.054 (1.014-1.096) p=0.008 | 1.084 (1.04-1.13) p<=0.001 | 1.17 (1.121-1.221) p<=0.001 | 1.168 (1.076-1.268) p<=0.001 |
| gram-positive bacteria | 1.007 (1.004-1.01) p<=0.001 | 1.073 (1.024-1.124) p=0.003 | 1.056 (1.004-1.112) p=0.036 | 1.124 (1.067-1.184) p<=0.001 | 1.13 (1.028-1.242) p=0.011 |
| CoNS | 1.011 (1.007-1.015) p<=0.001 | 1.114 (1.041-1.193) p=0.002 | 1.126 (1.045-1.214) p=0.002 | 1.211 (1.126-1.302) p<=0.001 | 1.193 (1.044-1.364) p=0.01 |
| Enterococcus spp. | 0.999 (0.994-1.004) p=0.724 | 1.065 (0.974-1.165) p=0.168 | 1.002 (0.906-1.109) p=0.964 | 1.004 (0.915-1.101) p=0.934 | 0.971 (0.813-1.159) p=0.745 |
| S.aureus | 1.011 (1.005-1.017) p<=0.001 | 1.03 (0.93-1.141) p=0.567 | 1.054 (0.954-1.163) p=0.299 | 1.192 (1.078-1.318) p<=0.001 | 1.259 (1.051-1.508) p=0.013 |
| Streptococcus spp. | 0.971 (0.948-0.995) p=0.017 | 1.155 (0.786-1.696) p=0.464 | 0.601 (0.348-1.039) p=0.068 | 0.69 (0.441-1.078) p=0.103 | 0.498 (0.173-1.43) p=0.195 |
| Corynebacteriaceae spp. | 1.000 (0.976-1.026) p=0.984 | 1.093 (0.678-1.763) p=0.714 | 1.112 (0.649-1.908) p=0.699 | 0.96 (0.598-1.544) p=0.868 | 1.274 (0.531-3.06) p=0.587 |
| gram-negative bacteria | 1.018 (1.013-1.023) p<=0.001 | 1.115 (1.028-1.209) p=0.009 | 1.216 (1.121-1.319) p<=0.001 | 1.329 (1.227-1.439) p<=0.001 | 1.378 (1.169-1.625) p<=0.001 |
| E. coli | 1.008 (0.998-1.018) p=0.105 | 1.151 (0.957-1.383) p=0.134 | 1.097 (0.913-1.319) p=0.324 | 1.166 (0.979-1.388) p=0.085 | 1.239 (0.875-1.754) p=0.228 |
| Klebsiella spp. | 1.012 (1.003-1.022) p=0.009 | 1.059 (0.889-1.262) p=0.523 | 1.105 (0.932-1.31) p=0.252 | 1.181 (1.002-1.393) p=0.048 | 1.616 (1.219-2.144) p<=0.001 |
| Enterobacter spp. | 1.038 (1.027-1.049) p<=0.001 | 1.2 (0.97-1.486) p=0.094 | 1.532 (1.246-1.884) p<=0.001 | 1.797 (1.471-2.196) p<=0.001 | 1.749 (1.215-2.518) p=0.003 |
| P. aeruginosa | 1.015 (1.003-1.028) p=0.012 | 1.069 (0.854-1.339) p=0.56 | 1.254 (1.006-1.564) p=0.044 | 1.335 (1.071-1.664) p=0.01 | 0.896 (0.538-1.494) p=0.675 |
| Serratia spp. | 1.009 (0.992-1.025) p=0.307 | 1.369 (1.024-1.828) p=0.034 | 1.393 (1.008-1.925) p=0.045 | 1.183 (0.859-1.63) p=0.303 | 1.079 (0.537-2.167) p=0.83 |
| Acinetobacter spp. | 1.042 (1.019-1.066) p<=0.001 | 1.532 (0.972-2.417) p=0.066 | 1.527 (0.962-2.424) p=0.072 | 1.93 (1.271-2.929) p=0.002 | 2.37 (1.178-4.771) p=0.016 |
| Proteus spp. | 1.015 (0.993-1.038) p=0.173 | 1.045 (0.695-1.573) p=0.832 | 1.07 (0.68-1.684) p=0.769 | 1.318 (0.915-1.898) p=0.138 | 1.283 (0.622-2.644) p=0.5 |
| S.maltophilia | 1.03 (1-1.06) p=0.046 | 0.678 (0.396-1.163) p=0.158 | 0.939 (0.547-1.61) p=0.818 | 1.303 (0.83-2.048) p=0.25 | 1.717 (0.843-3.496) p=0.136 |
| Citrobacter spp. | 1.02 (0.993-1.048) p=0.153 | 1.446 (0.851-2.457) p=0.172 | 1.521 (0.839-2.757) p=0.167 | 1.682 (0.999-2.83) p=0.05 | 0.637 (0.153-2.654) p=0.535 |
| Bacteroides spp. | 1.034 (1.002-1.067) p=0.035 | 0.988 (0.516-1.892) p=0.97 | 1.657 (0.902-3.043) p=0.104 | 1.602 (0.9-2.849) p=0.109 | 1.975 (0.776-5.029) p=0.154 |
| with fungi | 1.02 (1.011-1.029) p<=0.001 | 0.915 (0.783-1.068) p=0.259 | 1.125 (0.967-1.309) p=0.127 | 1.331 (1.144-1.549) p<=0.001 | 1.104 (0.819-1.49) p=0.516 |
| C.albicans | 1.019 (1.007-1.03) p=0.001 | 0.897 (0.75-1.073) p=0.235 | 1.088 (0.907-1.306) p=0.363 | 1.265 (1.054-1.518) p=0.012 | 1.221 (0.859-1.737) p=0.266 |
| other than albicans Candida | 1.022 (1.005-1.039) p=0.009 | 0.942 (0.68-1.305) p=0.721 | 1.262 (0.949-1.678) p=0.11 | 1.4 (1.035-1.894) p=0.029 | 0.883 (0.457-1.705) p=0.71 |
| other fungi | 1.017 (0.993-1.042) p=0.16 | 0.815 (0.509-1.305) p=0.395 | 0.95 (0.612-1.476) p=0.821 | 1.417 (0.947-2.12) p=0.09 | 0.653 (0.237-1.8) p=0.41 |

PBSI, primary bloodstream infection; CoNS, coagulase negative staphylococci. All multivariable GEE models are adjusted for length of stay, central venous catheter use, invasive ventilation, type of ICU, size of hospital, long-term trend and accounted for clustering.
